# Hyper-acute cardiovascular magnetic resonance T1 mapping predicts infarct characteristics in patients with ST elevation myocardial infarction

**DOI:** 10.1186/s12968-019-0593-9

**Published:** 2020-01-09

**Authors:** Mohammad Alkhalil, Alessandra Borlotti, Giovanni Luigi De Maria, Mathias Wolfrum, Sam Dawkins, Gregor Fahrni, Lisa Gaughran, Oxford Acute Myocardial Infarction (OxAMI) Study, Jeremy P. Langrish, Andrew Lucking, Vanessa M. Ferreira, Rajesh K. Kharbanda, Adrian P. Banning, Erica Dall’Armellina, Keith M. Channon, Robin P. Choudhury

**Affiliations:** 1 Acute Vascular Imaging Centre, Radcliffe Department of Medicine, University of Oxford, Oxford, UK; 2 Oxford Heart Centre, NIHR Biomedical Research Centre, Oxford University Hospitals, Oxford, UK; 3 Division of Cardiovascular Medicine, University of Oxford Centre for Clinical Magnetic Resonance Research (OCMR), Oxford, UK; 4 Leeds Institute of Cardiovascular and Metabolic Medicine, Department of Biomedical Imaging Sciences, University of Leeds, Leeds, UK; 5 Division of Cardiovascular Medicine, BHF Centre of Research Excellence, Radcliffe Department of Medicine, University of Oxford, Oxford, OX3 9DU UK

**Keywords:** STEMI, T1 mapping, CMR

## Abstract

**Background:**

Myocardial recovery after primary percutaneous coronary intervention in acute myocardial infarction is variable and the extent and severity of injury are difficult to predict. We sought to investigate the role of cardiovascular magnetic resonance T1 mapping in the determination of myocardial injury very early after treatment of ST-segment elevation myocardial infarction (STEMI).

**Methods:**

STEMI patients underwent 3 T cardiovascular magnetic resonance (CMR), within 3 h of primary percutaneous intervention (PPCI). T1 mapping determined the extent (area-at-risk as %left ventricle, AAR) and severity (average T1 values of AAR) of acute myocardial injury, and related these to late gadolinium enhancement (LGE), and microvascular obstruction (MVO). The characteristics of myocardial injury within 3 h was compared with changes at 24-h to predict final infarct size.

**Results:**

Forty patients were included in this study. Patients with average T1 values of AAR ≥1400 ms within 3 h of PPCI had larger LGE at 24-h (33% ±14 vs. 18% ±10, *P* = 0.003) and at 6-months (27% ±9 vs. 12% ±9; *P* < 0.001), higher incidence and larger extent of MVO (85% vs. 40%, *P* = 0.016) & [4.0 (0.5–9.5)% vs. 0 (0–3.0)%, *P* = 0.025]. The average T1 value was an independent predictor of acute LGE (*β* 0.61, 95%CI 0.13 to 1.09; *P* = 0.015), extent of MVO (*β* 0.22, 95%CI 0.03 to 0.41, *P* = 0.028) and final infarct size (*β* 0.63, 95%CI 0.21 to 1.05; *P* = 0.005). Receiver-operating-characteristic analysis showed that T1 value of AAR obtained within 3-h, but not at 24-h, predicted large infarct size (LGE > 9.5%) with 100% positive predictive value at the optimal cut-off of 1400 ms (area-under-the-curve, AUC 0.88, *P* = 0.006).

**Conclusion:**

Hyper-acute T1 values of the AAR (within 3 h post PPCI, but not 24 h) predict a larger extent of MVO and infarct size at both 24 h and 6 months follow-up. Delayed CMR scanning for 24 h could not substitute the significant value of hyper-acute average T1 in determining infarct characteristics.

## Background

One year mortality remains high at ~ 10% in patients presenting with ST-segment elevation myocardial infarction (STEMI), despite emergency revascularisation by primary percutaneous coronary intervention (PPCI) [1]. Early recognition of a high-risk subgroup may enable patient selection for specific therapies to improve early outcomes and reduce future risk [1, 2]. Existing approaches rely on recognition of adverse outcomes after the acute phase of STEMI, when myocardial injury is irreversible, are largely based on treating the “average” patient and do not reflect tissue characteristics at an individual patient level [1, 2].

Cardiovascular magnetic resonance (CMR) is widely used to characterise injured myocardium [3]. Ischaemic and infarcted tissues are affected by oedema, necrosis, haemorrhage and disruption of microvascular integrity [3]. This complex tissue-level heterogeneity makes CMR appealing for non-invasive characterisation of myocardial injury after myocardial infarction [3]. Previous studies have demonstrated the utility of undertaking CMR imaging 24–72 h after PPCI, and some as early as few hours after myocardial infarction (MI) [4–6]. These studies revealed imaging features most likely reflective of the underlying dynamic tissue-level changes [4, 5]. Late gadolinium enhancement (LGE) performed within 12 h of STEMI is predictive of future adverse events beyond traditional clinical risk factors such as age and diabetes [7]. Whilst the quantification of myocardial injury depends on the volume of myocardium subtended by the artery beyond its occlusion and the ischaemia time, other factors may have a significant impact on myocardial recovery beyond the initial volumetric injury, such as the duration and persistence of occlusion, the contribution of collateral vessels, the effects of embolic debris, release of vasoactive substances, reperfusion injury, and myocardial oedema [8]. In a given patient, these individual variables cannot currently be ascertained with accuracy, nor can their effects be discerned in isolation. We hypothesized that the integrated effect of these many factors would be manifest very early (< 3 h) after PPCI by direct tissue-level assessment using CMR, before the ‘secondary’ processes in myocardial injury, such a reactive oedema, had supervened [5, 6, 9]. Likewise, these early changes may shed more light into the development of certain pathophysiological processes within the infarcted region such as the development and progression of microvascular obstruction and haemorrhage. An advantage of such an approach would be the potential to stratify patients to specific treatment pathways early after PPCI, before irreversible myocardial injury had occured [3].

Recently, native T1 mapping was demonstrated to reflect tissue composition, with changes in T1 relaxation times reflecting pathological processes at the level of myocardial tissue [10, 11]. Moreover, T1 mapping can characterise injured myocardium, allowing assessments of both severity of injury and potential for recovery [4, 10], making this technique ideally suited to the evaluation and quantification of myocardial injury after STEMI.

Accordingly, we designed a proof-of-concept study using CMR T1 mapping for myocardial tissue characterisation, to test whether very early CMR imaging, within 3 h after PPCI, could provide prospective useful information to predict the evolution of myocardial injury, and final infarct size, in STEMI patients.

## Methods

### Study population

Patients presenting with STEMI to the John Radcliffe Hospital, Oxford who underwent PPCI for an occluded coronary artery were prospectively enrolled as part of the Oxford Acute Myocardial Infarction (OxAMI) project (see Additional file 1) [4, 12]. This was a pre-specified study within the OxAMI research programme, and patients were prospectively recruited. These data have not been reported in any other OxAMI published studies [4, 12]. The study protocol was approved by the local research ethics committee and conducted in accordance with the Declaration of Helsinki. All participants provided initial verbal assent followed by written informed consent, in accordance with the approved study protocol.

### Cardiac magnetic resonance protocol

3 T CMR was performed (MAGNETOM Verio, Siemens Healthineers, Erlangen, Germany) within 3 h of stent implantation (hyper-acute), at 24 h (acute) and at 6 months (follow-up). The scan protocol comprised cine balanced steady state free precession (bSSFP) for functional images, native T1 mapping using the shortened modified Look-Locker inversion recovery (ShMOLLI) for characterisation of area-at-risk (AAR) [4, 11], T2* mapping for intra-myocardial haemorrhage (IMH), and late gadolinium enhancement (LGE).

Typical acquisition parameters for bSSFP retrospectively gated cine images were TE / TR =1.4/3.2 ms; flip angle 50°; voxel size: 2.4 × 1.8 × 8.0 mm. To shorten the hyper-acute scan, the cine sequence at TP1 were acquired in a single breath hold using real time electrocardiogram (ECG)-triggered and at lower resolution (160 × 72) compared to other time points (224 × 137).

T2 weighted (T2w) was performed using a T2-prep-bSSFP single shot sequence with surface coil correction (TE/TR = 1/4.1 msec; effective TE = 60 msec; flip angle 90°; voxel size: 2.1 × 1.6 × 8.0 mm).

ShMOLLI T1 maps were generated from 5 to 7 bSSFP images with variable inversion preparation S2 time as described previously [11]. Typical acquisition parameters were: TE/TR = 1.07/2.14 msec, flip angle = 35°, FOV = 340 × 255 mm, matrix size = 192 × 144, 107 phase encoding steps, actual experimental voxel size = 1.8 × 1.8 × 8 mm, interpolated reconstructed voxel size = 0.9 × 0.9 × 8 mm, GRAPPA = 2, 24 reference lines, cardiac delay time TD = 500 msec and 206 msec acquisition time for single image, phase partial Fourier 6/8.

T2* maps were obtained using a gradient echo sequence. Typical imaging parameters were: TR 600 ms, echo numbers (*n* = 5), TE 22.14 ms, FOV 340 × 225 mm, bandwidth 5260 Hz/Px, matrix = 192 × 144, voxel size = 1.8 × 1.8 × 3.0 mm, flip angle 20°.

LGE was performed with a T1- weighted segmented inversion recovery gradient echo-phase sensitive-inversion recovery (GRE PSIR) sequence (TE/TR = 2.5 msec/5 msec, voxel size =1.8 × 1.4 × 8.0 mm, flip angle 40°). LGE images were collected 10–15 min after the administration of 0.1 mmol/kg contrast agent (Dotarem, Guerbet, Villepinte, France) [4]. The inversion time was adjusted for optimal nulling of remote normal myocardium.

Hyper-acute T1 mapping was obtained from three short axis slices targeting the regional wall motion abnormalities as assessed on functional images. This protocol was specifically designed to minimise delays in admission to the coronary care unit, given the hyper-acute clinical setting of performing CMR immediately after PPCI for STEMI.

### CMR imaging analysis

**Fig. 1:**
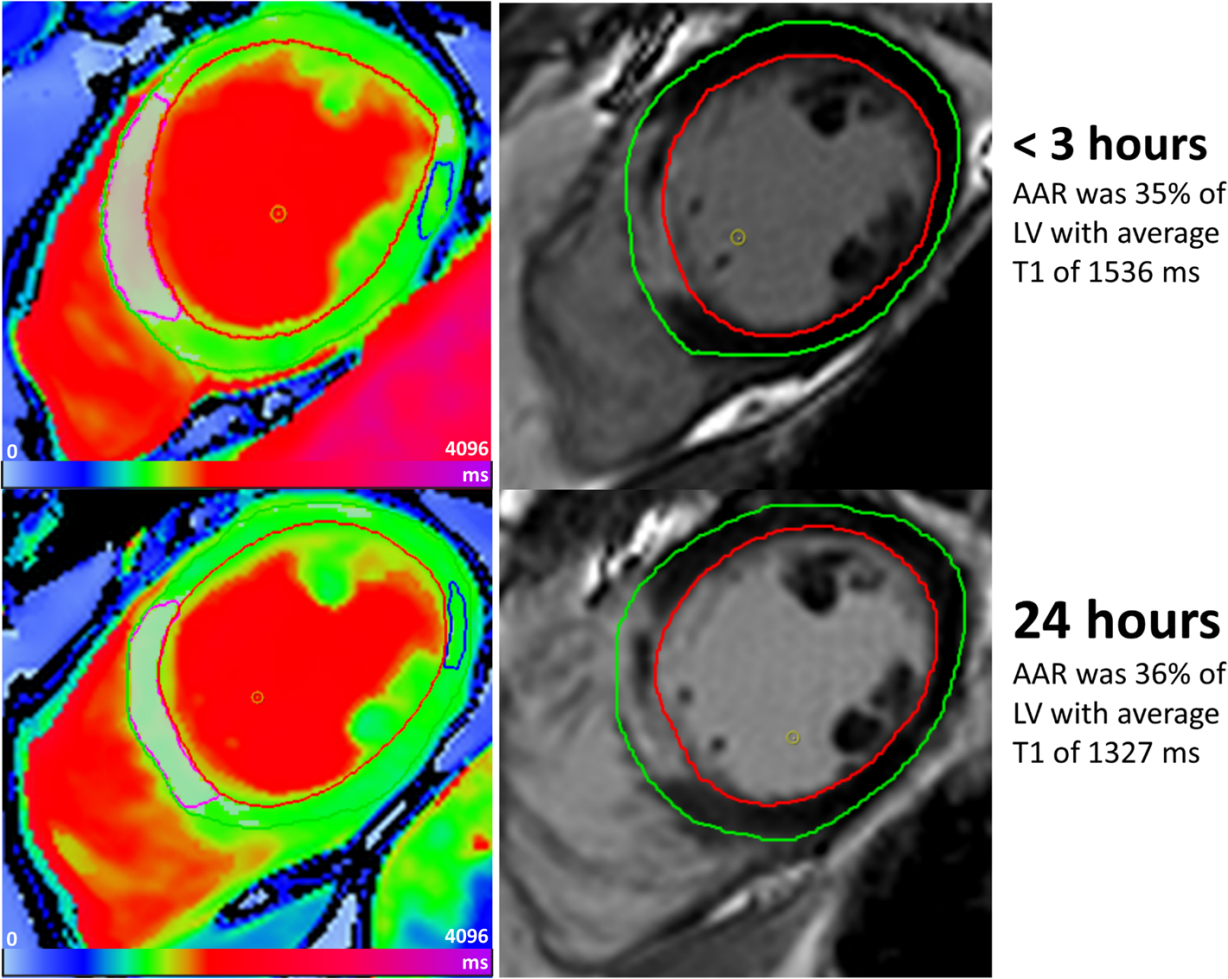
Identification of area at risk (AAR) using cardiovascular magnetic resonance (CMR) T1 mapping. AAR was automatically delineated (pink contour) using threshold of 2SD above the mean value of remote reference region of interest (ROI, contoured in blue) placed 180 degrees opposite to the injured myocardium. This process was performed irrespective of presence of microvascular obstruction (MVO)

Cvi42 image analysis software (Circle Cardiovascular Imaging Inc., Calgary, Canada) was used by two experienced operators in CMR image analysis. Left ventricular (LV) volumes and ejection fraction (EF) were assessed from cine bSSFP images. AAR on T1 mapping was identified similarly to previous reports using a threshold of 2SD above the mean value of remote reference region of interest (ROI) placed 180 degrees opposite to the injured myocardium with no visible regional wall abnormalities or infarction (assessed by inspecting corresponding cine and LGE images, respectively) [13]. T1 values of the delineated area were subsequently averaged to assess the severity of myocardial oedema, irrespective of the presence of microvascular obstruction (MVO) (Fig. 1); additionally, this area was measured as a percentage of the LV mass (AAR).

LGE was depicted using signal intensity threshold set at 5 standard deviations above the remote reference myocardium [12]. When present, MVO was included in the measurements of LGE. The LV MVO percentage fraction was quantified by manual delineation of the hypointense areas within the LGE region [12]. IMH was defined on T2* maps as a hypointense area within the injured myocardium having a mean signal intensity 2SD below the signal intensity of the periphery of oedematous region and a mean T2* value < 20 ms. Myocardial salvage index (MSI) was calculated by subtracting 6 months %infarct size from 24 h %myocardial oedema (derived from T1 mapping) and then divided by %myocardial oedema at 24 h as previously described [4].

Patients with average T1 values > 1400 ms within AAR were defined as high T1 value group. This threshold was previously reported to discriminate reversible versus irreversible myocardial injury using the same 3 T CMR scanning platform and T1-mapping technique [4]. ‘Large’ infarcts at 6 months were defined using a previously published cut-off of final infarct size of 9.5%, derived from LGE, as a surrogate of long term clinical outcomes [14].

### Angiographic and electrocardiographic analysis

Angiographic analyses were performed offline by two experienced operators blinded to CMR parameters and cases of disagreement were resolved by consensus. Final thrombolysis in myocardial infarction (TIMI) flow and post-procedural myocardial blush grade (MBG) were recorded [15]. Angiographic thrombus burden was graded as previously described [15], and patients with thrombus score of 5 were considered to have large thrombus burden. Bystander coronary artery disease (CAD) in the non-culprit artery was defined as > 50% luminal stenosis, measured by two dimensional quantitative coronary angiography (Medcon QCA software, Medcon Limited, Tel Aviv, Israel) as previously described [12, 15].

A 12-lead ECG was recorded at admission and 60 min after PPCI in all patients and ST resolution was defined as more than 70% reduction in sum of ST-segments (see Additional file 1).

### Statistical analysis

Normality of distribution was assessed using the Shapiro-Wilk test. All variables are expressed as mean ± standard deviation or as median (IQR; interquartile range) as appropriate. Frequency comparisons were made using Chi-squared test or Fisher’s exact test, as appropriate, whilst continuous variables were compared by using unpaired Student’s t-test for parametric data or Mann-Whitney U test for non-parametric data. A multivariate regression model was constructed to determine if T1 value is an independent predictor of CMR measurement (for every 10 ms increase in T1 values) after adjustment for available clinical and angiographic characteristics within 3 h of STEMI presentation. In order not to overload the model, only variables with *P* < 0.05 on univariate analysis were entered. These variables included age, gender, diabetes and hypertension status, mean blood pressure at presentation, location of infarct (anterior versus non-anterior) stent length and diameter, use of glycoprotein IIb/IIIa inhibitors, TIMI and myocardial blush grade at the end of procedure, thrombus score, ST segment resolution, ischaemia time, door-to-balloon time, troponin value, in addition to the extent of injury as LV% (AAR) (no multi-collinearity was detected on any model). All statistical analyses were performed using SPSS 22.0 (Statistical Package for the Social Sciences (SSPS), International Business Machines, Inc., Armonk, New York, USA) and a *P* value < 0.05 was considered statistically significant.

## Results

**Fig. 2:**
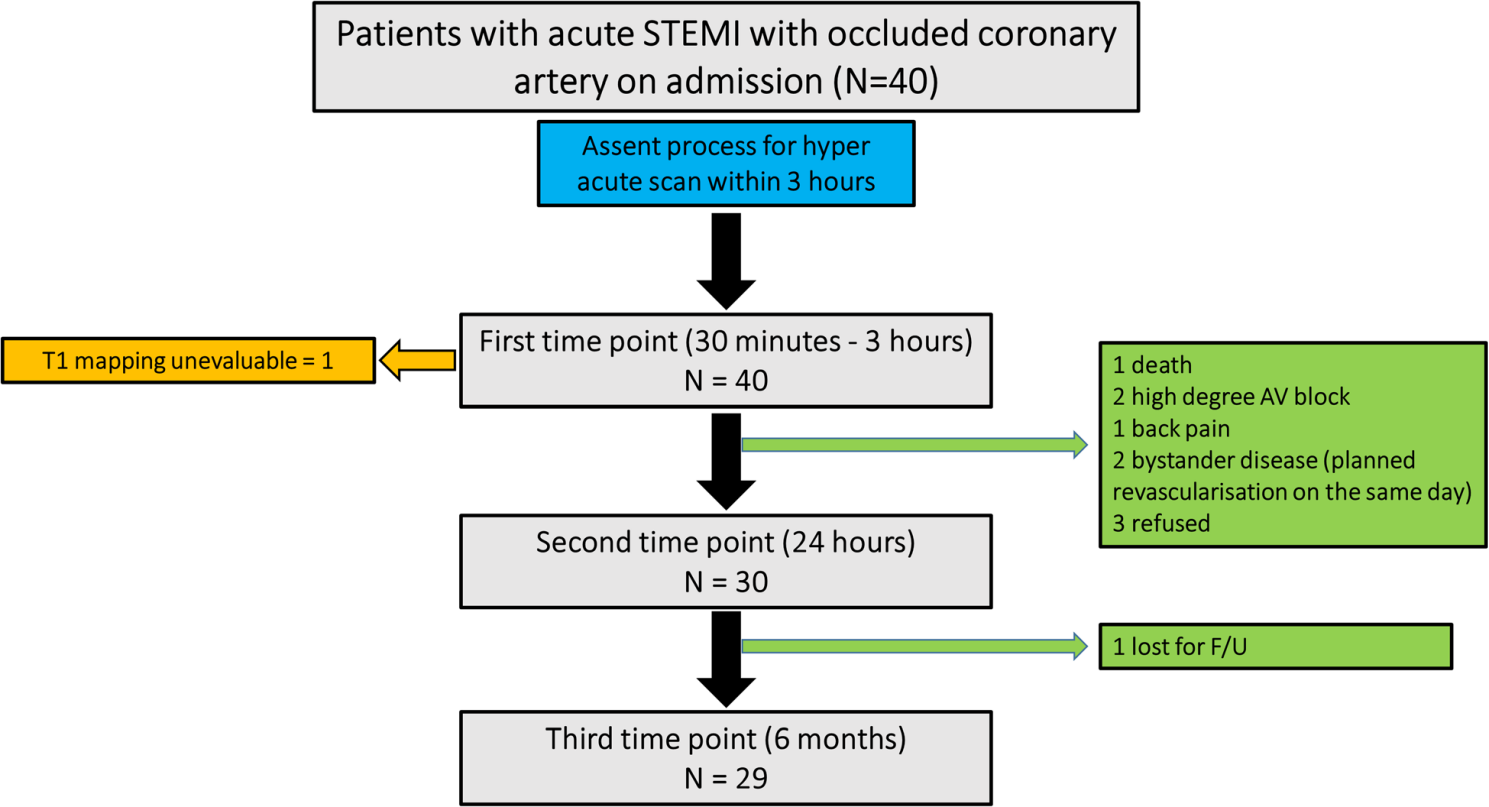
Study flow chart. Patients presenting with ST elevation myocardial infarction (STEMI) & occluded vessel were prospectively enrolled to have a hyper-acute CMR imaging (within 3 h)

Forty STEMI patients (62 ± 11 years; 32 (82%) male) underwent hyper-acute CMR within 3 h of PPCI. Thirty (75%) patients were scanned at 24 h (acute) and 29 (73%) patients came back for the 6-month follow-up scan (Fig. 2). One patient had unevaluable T1 mapping at the index CMR scan. The median ischemia time, defined from onset of chest pain until restoration of coronary blood flow, was 183 min (IQR 153–301). Hyper-acute CMR scans were performed at 122 ± 55 mins after PPCI (median 112, IQR 73–151). The average duration of CMR scanning was 33 ± 6 min.

### Role of hyper-acute T1 value within AAR

There were no differences in clinical and procedural characteristics, or time to CMR scanning between subjects with AAR-T1 values ≥ or < 1400 ms (Table 1).

**Table 1:** Clinical characteristics of recruited patients stratified by average T1 values within the area at risk (AAR) at 3 h after primary percutaneous coronary intervention (PPCI)

Clinical characteristics	Whole cohort	AAR with average T1 value (< 1400 ms) (*n* = 24)	AAR with average T1 value (≥1400 ms) (*n* = 15)	*P* value
Age^a^	62 ± 11	61 ± 12	62 ± 10	0.88
Male gender^b^	32 (82)	18 (75)	14 (93)	0.22
Body Surface Area^a^	2.06 ± 0.20	2.04 ± 0.19	2.10 ± 0.22	0.35
Hypertension^b^	15 (38)	11 (46)	4 (27)	0.23
Dyslipidaemia^b^	12 (31)	8 (33)	4 (26)	0.66
Active smoking^b^	13 (33)	8 (33)	5 (33)	1.00
Diabetes^b^	3 (8)	3 (13)	0	0.27
Ischaemia time (mins)^c^	183 (153–301)	166 (148–269)	229 (182–352)	0.09
Door-to-balloon time (mins)^c^	27 (18–43)	24 (14–38)	31 (24–66)	0.16
Systolic pressure (mmHg)^a^	126 ± 27	130 ± 25	118 ± 29	0.19
Diastolic pressure (mmHg)^a^	73 ± 16	73 ± 15	73 ± 18	0.93
Anterior infarct^b^	11 (28)	5 (21)	6 (40)	0.20
Number of diseased vessels^c^	1 (1–2)	1 (1–2)	1 (1–2)	0.94
Bystander disease^b^	15 (38%)	9 (38%)	6 (40%)	0.88
Large thrombus burden^b^	18 (46%)	10 (42%)	8 (53%)	0.48
Thrombectomy use^b^	22 (56)	14 (58)	8 (53)	0.76
GP IIb/IIIa^b^	6 (27)	3 (13)	3 (20)	0.66
Stent length (mm)^a^	31 ± 13	34 ± 15	26 ± 8	0.07
Stent diameter (mm)^c^	3.5 (3.0–4.0)	3.5 (3.1–4.0)	3.5 (3.0–4.0)	0.24
Final TIMI III flow^b^	28 (72)	19 (79)	9 (60)	0.20
MBG 2/3^b^	24 (62%)	16 (67%)	8 (53%)	0.41
Time to CMR (mins)^a^	122 ± 55	120 ± 58	125 ± 51	0.79
ST resolution^b^	9 (23%)	5 (21%)	4 (27%)	0.67

Large thrombus burden was defined thrombus score ≥ 4. There was no difference in baseline clinical and procedural characteristics in those with or without 6 months follow up

^a^(mean ± SD), ^b^(n, %), ^c^ (median, IQR)

There were significant differences in acute (24 h) CMR parameters using the pre-specified 1400 ms T1 cut-off obtained at the hyper-acute scan. Patients with hyper-acute T1>1400 ms in the injured myocardium had larger LV end diastolic volume (LVEDV) (184 ± 30 vs. 154 ± 34 ml; *P* = 0.021), LGE myocardium (33% ±14 vs. 18% ±10; *P* = 0.003), MVO incidence (85% vs. 40%; *P* = 0.016) and extent of MVO [4.0 (0.5–9.5)% vs. 0 (0–3.0)%, *P* = 0.025] (Table 2). These patients tended to have larger LV systolic volume (LVESV), incidence and extent of IMH, but these did not reach statistical difference. There was no difference in LV ejection fraction between two groups (46% ±10 vs. 49% ±11; *P* = 0.44), or regression in extent of LGE (7.3 ± 6.5% vs. 4.5 ± 3.8%, *P* = 0.12).

**Table 2:** Acute (24 h) CMR characteristics stratified by average T1 values within the AAR at 3 h after PPCI

Acute CMR characteristics	Whole cohort	AAR with average T1 value (< 1400 ms) (*n* = 17)	AAR with average T1 value (≥ 1400 ms) (*n* = 13)	*P* value
LV end diastolic volume *(ml)*^b^	168 ± 35	154 ± 34	184 ± 30	0.021
LV end systolic volume *(ml)*^b^	89 ± 31	80 ± 33	99 ± 26	0.103
LV ejection fraction *(%)*^b^	48 ± 10	49 ± 11	46 ± 10	0.442
Area at risk *(%)*^b^	40 ± 12	34 ± 6	48 ± 12	0.002
LGE myocardium *(%)*^b^	25 ± 14	18 ± 10	33 ± 14	0.003
MVO incidence^a, c^	17 (61)	6 (40)	11 (85)	0.016
MVO extent *(%)*^d^	1 (0–6.5)	0 (0–3.0)	4.0 (0.5–9.5)	0.025
IMH incidence^a, c^	15 (54)	6 (40)	9 (69)	0.122
IMH extent *(mm*^*2*^*)*^d^	0.76 (0–2.03)	0 (0–1.40)	0.76 (0–4.21)	0.152

*IMH* Intramyocardial haemorrhage, *LGE* Late gadolinium enhancement, *LV* Left ventricular, *MVO* Microvascular obstruction

^a^Two patients with poor LGE and T2* mapping images were excluded from the analysis. ^b^(mean ± SD), ^c^(n, %), ^d^(median, IQR)

**Fig. 3:**
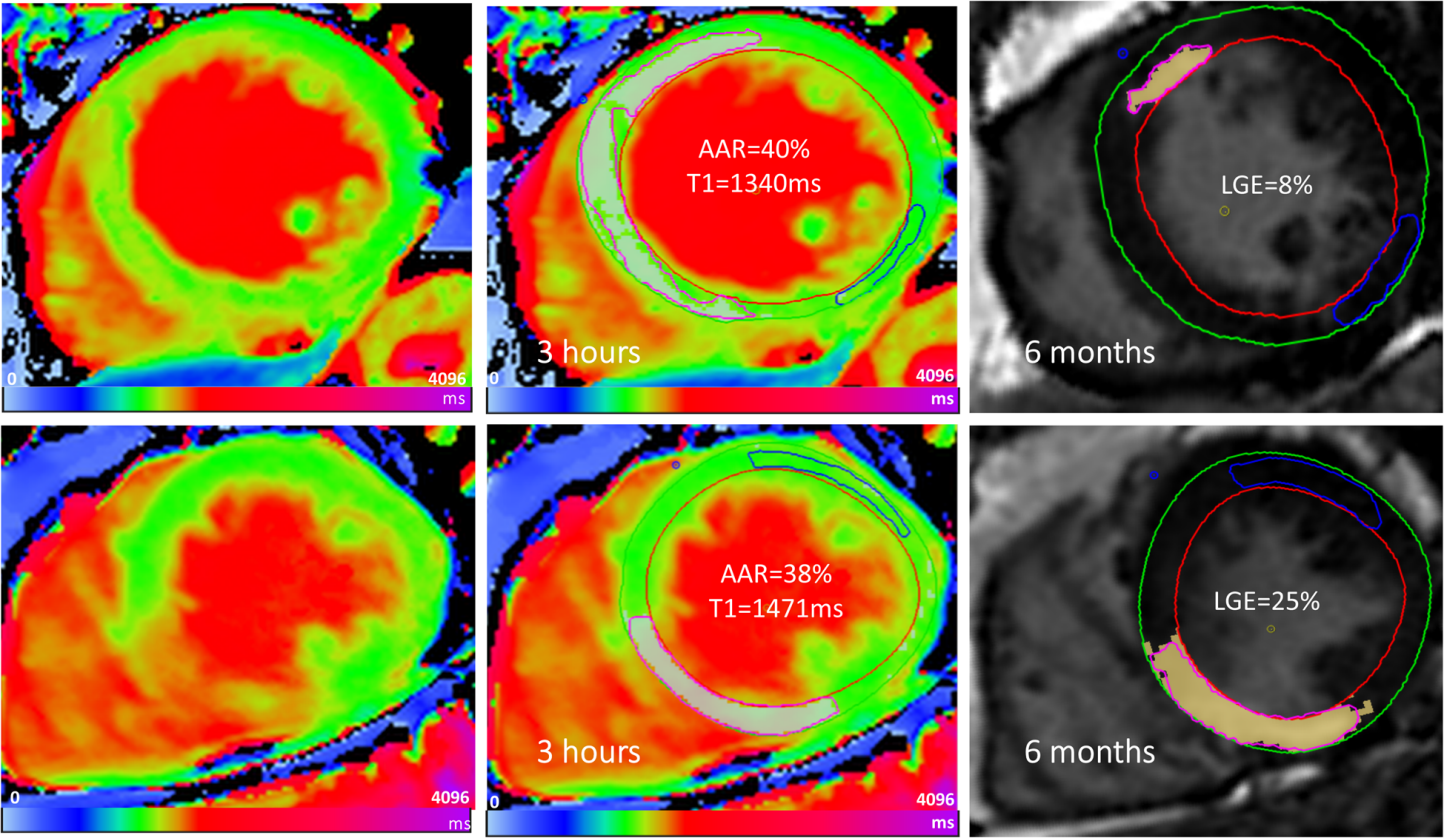
Myocardial T1 value and AAR to predict final infarct size. An example of two patient presenting with anterior STEMI with comparable AAR. Hyper-acute T1 mapping was used to quantify AAR and infarct severity. Despite a relatively similar AAR (LV%), patients with less-elevated average T1 value within the AAR (< 1400 ms; top row) had smaller final infarct sizes at 6 months compared to patients with higher T1 values within the AAR (≥1400 ms)

Similarly, LVEDV at follow-up CMR was larger (197 ± 49 vs. 159 ± 26 ml; *P* = 0.020) with a trend towards larger LVESV (99 ± 40 vs. 74 ± 19 ml, *P* = 0.050) in patients with hyper-acute T1 values of AAR > 1400 ms. Final infarct size (27% ±9 vs. 12% ±9, *P* < 0.001) was also larger while the myocardial salvage index was smaller (45% (37–54) vs. 71% (47–90), *P* = 0.021) (Table 3 and Fig. 3).

**Table 3:** CMR characteristics at 6 months stratified by average T1 values within the AAR at 3 h after PPCI

FU CMR characteristics	Whole cohort	AAR with average T1 value (< 1400 ms) (*n* = 16)	AAR with average T1 value (≥ 1400 ms) (*n* = 13)	*P* value
LV end diastolic volume *(ml)*^a^	175 ± 42	159 ± 26	197 ± 49	0.020
LV end systolic volume *(ml)*^a^	86 ± 32	74 ± 19	99 ± 40	0.050
LV ejection fraction *(%)*^a^	52 ± 8	53 ± 9	51 ± 8	0.494
MSI *(%)*^b^	50 (39–71)	71 (47–90)	45 (37–54)	0.021
Final infarct size *(%)*^a^	19 ± 11	12 ± 9	27 ± 9	< 0.001
Large infarct^c^	20 (69%)	7 (44%)	13 (100%)	0.001

^a^(mean ± SD), ^b^ (median, IQR), ^c^(n, %)

Clinical features including age, gender, diabetes status, infarct location, blood pressure on arrival, ischaemia time, Killip-class on admission, or ECG parameters such as presence of Q wave and QRS duration did not predict infarct severity as determined by average T1 mapping. Similarly, none of procedural characteristics including thrombus score, use of glycoprotein IIb/IIIa inhibitors, stent diameter and length reached statistical significance to ascertain association with infarct severity.

At the hyper-acute time point, there was a significant relationship between average T1 value and AAR (r = 0.66, *P* < 0.001).

### Hyper-acute extent of injury (AAR) as predictors of acute and follow-up CMR measurements

Univariate regression analysis revealed that hyper-acute AAR was a predictor of acute LV volumes, extent of LGE, MVO and IMH, in addition to LV ejection fraction (Table 4). However, this association was lost when adding other significant predictors such as the angiographic-derived thrombus score and myocardial blush grade for MVO and IMH extent. Hyper-acute AAR remained a significant predictor of acute LV volumes [EDV (*β* 1.21, 95% CI 0.15 to 2.28, *P* = 0.027) and ESV (*β* 0.94, 95% CI 0.02 to 1.86, *P* = 0.046)], ejection fraction (*β* − 0.31, 95% CI − 0.56 to − 0.07, *P* = 0.015) and LGE (*β* 0.39, 95% CI 0.04 to 0.74, *P* = 0.029) (Table 4).

**Table 4:** AAR (%LV) as a predictor of acute and follow-up CMR outcomes

Extent of injury		Univariate regression analysis			Multivariate regression analysis^b^		
		*β* Coefficient	95% CI	*P* value	*β* Coefficient	95% CI	*P* value
Acute CMR (24 h)	EDV	1.21	0.15,2.28	0.027	1.21^a^	0.15,2.28	0.027
	ESV	1.53	0.72,2.34	0.001	0.94	0.02,1.86	0.046
	EF	−0.57	−0.81,-0.32	< 0.001	− 0.31	− 0.56,− 0.07	0.015
	LGE%	0.76	0.39,1.12	< 0.001	0.39	0.04,0.74	0.029
	MVO extent	0.23	0.07,0.38	0.006	0.07	-0.07,0.20	0.336
	IMH extent	0.07	0,0.15	0.039	0.03	−0.04,0.11	0.362
Follow-up CMR (6 months)	EDV	1.23	0.04,2.42	0.043	0.54	−0.66,1.74	0.361
	ESV	1.24	0.39,2.08	0.006	0.86	0.04,1.67	0.041
	EF	−0.32	−0.53,-0.1	0.006	−0.27	−0.51,-0.04	0.023
	MSI	−0.88	−1.52,-0.23	0.010	−0.43	−1.15,0.30	0.235
	Final infarct size	0.57	0.32,0.83	< 0.001	0.47	0.22,0.72	0.001

^a^None of the variables in the model was a predictor of EDV (average T1 value of AAR was not included), ^b^For all CMR outcomes, adjustment was made for the following variables: age, gender, diabetes and hypertension status, mean blood pressure at presentation, location of infarct (anterior versus non-anterior) stent length and diameter, use of glycoprotein IIb/IIIa inhibitors, TIMI and myocardial blush grade at the end of procedure, thrombus score, ST segment resolution, ischaemia time

Similarly, hyper-acute AAR was a predictor of follow-up LV volumes, ejection fraction, MSI and final infarct size on univariate regression analysis. This prediction remained significant for follow-up CMR measurements except for LVEDV and MSI, when analysed by multivariate regression analysis LVESV (*β* 0.86, 95% CI 0.04 to 1.67, *P* = 0.041), ejection fraction (*β* − 0.27, 95% CI − 0.51 to − 0.04, *P* = 0.023), final infarct size (*β* 0.47, 95% CI 0.22 to 0.72, *P* = 0.001) (Table 4).

### Hyper-acute average T1 values of AAR as predictors of acute and follow-up CMR measurements

Hyper-acute average T1 *value* of the AAR was a predictor of acute LV volumes, and the extent of LGE, MVO and IMH on univariate regression analysis (Table 5). On multivariate regression analysis (after including AAR as LV%), the average T1 of AAR value was an independent predictor of LGE myocardium (*β* 0.61, 95% CI 0.13 to 1.09; *P* = 0.015) and extent of MVO (*β* 0.22, 95% CI 0.03 to 0.41; *P* = 0.028) (Table 5). The average T1 value of AAR was not an independent predictor of LVEDV or extent of IMH. Importantly, adding the average T1 value of AAR to the model rendered AAR (LV%) not significant in predicting acute LVEDV (*β* 0.78, 95% CI − 0.53 to 2.09, *P* = 0.23) or LGE (*β* 0.15, 95% CI − 0.21 to 0.52, *P* = 0.39).

**Table 5:** Hyper-acute average T1 value of injured myocardium as a predictor of acute and follow-up CMR outcomes

Severity of injury		Univariate regression analysis			Multivariate regression analysis^a^		
		*Β* Coefficient	95% CI	*P* value	*β* Coefficient	95% CI	*P* value
Acute CMR (24 h)	EDV	1.70	0.18,3.22	0.030	1.05	−0.82,2.91	0.260
	ESV	1.34	−0.01,2.68	0.052	–	–	–
	EF	−0.34	−0.80,0.12	0.141	–	–	–
	LGE%	1.06	0.54,1.59	< 0.001	0.61	0.13,1.09	0.015
	MVO extent	0.35	0.14,0.57	0.002	0.22	0.03,0.41	0.028
	IMH extent	0.11	0.01,0.21	0.031	0.08	−0.04,0.19	0.185
Follow-up CMR (6 months)	EDV	3.00	1.32,4.47	0.001	3.00	0.60,5.37	0.017
	ESV	2.32	1.15,3.51	< 0.001	1.76	0.35,3.17	0.016
	EF	−0.41	−0.78,-0.03	0.035	−0.09	−0.55,0.36	0.681
	MSI	−1.5	−2.41,-0.60	0.002	−1.13	−2.06,-0.19	0.021
	Final infarct size	0.99	0.62,1.36	< 0.001	0.65	0.25,1.05	0.003

^a^For all CMR outcomes, adjustment was made for the following variables: age, gender, diabetes and hypertension status, mean blood pressure at presentation, location of infarct (anterior versus non-anterior) stent length and diameter, use of glycoprotein IIb/IIIa inhibitors, TIMI and myocardial blush grade at the end of procedure, thrombus score, ST segment resolution, ischaemia time, door-to-balloon time, troponin value, in addition to the extent of injury as LV% (AAR)

Hyper-acute average T1 value of the AAR predicted follow-up LV volumes, EF, MSI and final infarct size (Table 5). On multivariate regression analysis, the average T1 value of AAR was an independent predictor of follow-up LV volumes (*β* 3.07, 95% CI 1.11 to 5.02; *P* = 0.003 for LVEDV) and (*β* 1.76, 95% 0.35 to 3.17; *P* = 0.016 for LVESV), MSI (*β* − 1.13, 95% CI − 2.06 to − 0.19; *P* = 0.021) and final infarct size (*β* 0.65, 95% CI 0.25, 1.05; P = 0.003) (Table 5). Remarkably, AAR (LV%) did not remain significant when adding average T1 value of AAR to the model in predicting LVESV (*β* 0.16, 95% CI − 0.78 to 1.09, *P* = 0.73), ejection fraction (*β* − 0.24, 95% CI − 0.54 to 0.07, *P* = 0.12) or final infarct size (*β* 0.21, 95% CI − 0.05 to 0.48, *P* = 0.11) at 6 months follow-up.

### T1 value threshold to predict large final infarct size

**Fig. 4:**
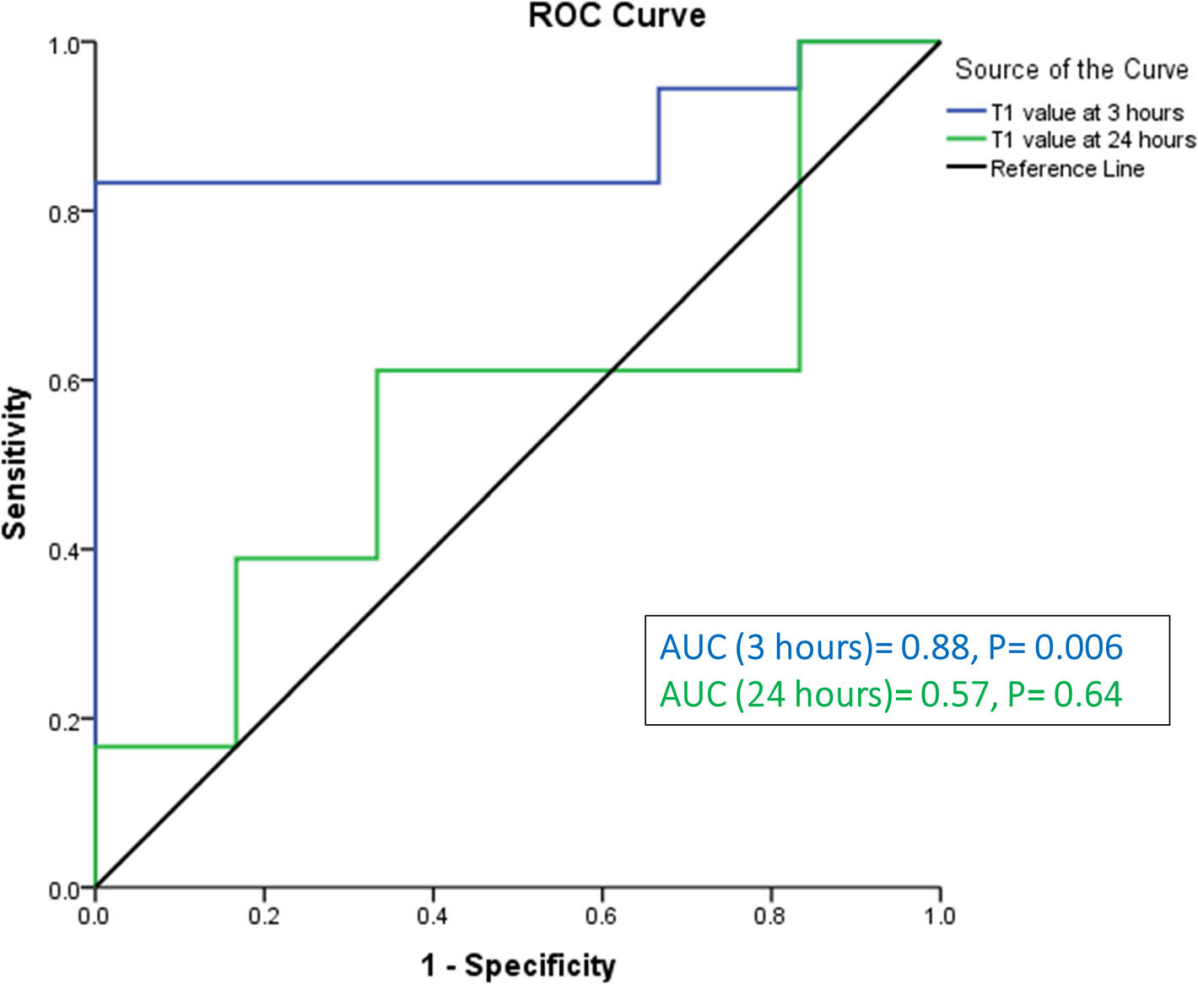
The ability of hyper-acute and acute average T1 value of AAR to predict large infarct size. The ability of using T1 values within the AAR to predict large infarct size was related to the hyper-acute timing of performing CMR imaging (within 3 h post-primary percutaneous coronary intervention (PPCI)). At 24 h, T1 value of AAR was not a predictor of large infarct size

Using the previously published cut-off of follow-up infarct size of 9.5% as a surrogate of long term clinical outcomes [14], receiver-operating characteristics (ROC) analysis using hyper-acute average T1 values of AAR (at 3 h post PPCI) demonstrated an area under curve (AUC) = 0.88, *P* = 0.006. By contrast, acute average T1 values of AAR (at 24 h post PPCI) did not predict large follow-up infarct size (AUC = 0.57, *P* = 0.64) (Fig. 4).

The optimal threshold for the hyper-acute average T1 value of AAR to predict large infarct size was 1396 ms, which was very similar to the value used to divide this cohort based on a previously published value of 1400 ms. [4] Using this threshold, average T1 value had a positive predictive value of 100% to predict large infarct size while its negative predictive value was 60%. The specificity and sensitivity of this threshold were 100 and 70%, respectively.

## Discussion

This is the first prospective study to evaluate the value of determining the severity of injured myocardium at a very early time point (median time < 2 h after reperfusion) in STEMI patients undergoing PPCI. The key findings of this study are: (1) Hyper-acute average T1 values of AAR were associated with infarct characteristics, whereby patients with higher average T1 values (> 1400 ms) had a larger extent of MVO and larger infarct size at both 24 h and 6 months follow-up; (2) hyper-acute average T1 value within the AAR, but not the extent (LV%) of AAR, independently predicted acute LGE myocardium and final infarct size at 6 months; (3) the significant value of hyper-acute average T1 value of AAR could not be substituted by delaying scan for 24 h and the utility of average T1 value exists only when CMR is performed at very early time point post STEMI.

Tools to identify patients at increased risk post STEMI are needed for prognostic and therapeutic purposes, in addition, to guide clinical pathways and safe resource allocation [1]. Our study demonstrated the potential role of hyper-acute T1 values as a determinant of infarct characteristics and a predictor of large infarct size during both the acute and follow up settings. Importantly, the role of T1 values was related to the timing of CMR imaging, which was performed immediately following reperfusion therapy following PPCI (median time < 2 h). The higher the average T1 value of the AAR, the more likely the patient was to sustain more severe injury irrespective of the initial volume of injury. The hyper-acute phase post STEMI is critical in the evolution of acute MI and early characterisation may allow for future selection of specific targeted therapies [3]. T1 mapping could provide an opportunity to risk stratify patients according to their predicted final infarct size at a very early stage enabling patient selection for trials to precisely assess the effectiveness of new interventions.

Notably, the ability of T1 mapping to differentiate high risk patients did not persist following the hyper-acute time point. Indeed, T1 value of AAR did not predict acute infarct size when obtained 24 h post STEMI. This may be related to inter-individual variations in the response to acute ischaemia and reperfusion, in addition to the dynamic changes of the severity of injured myocardium post reperfusion therapy [5, 9]. Human and animal models have demonstrated that the severity of injured myocardium has a bimodal phenomenon with the nadir at 24 h [5, 6, 9]. Furthermore, the emergence of an infarct core, detected using T1 mapping may have also contributed to the varied ability of using T1 values alone to assess the severity and prognosis of acute myocardial injury [13]. The hypo-intense infarct core identified using T1 mapping (typically seen as a “step-down” in T1 values within the AAR) was reported in 56% of patients after 1–2 days post STEMI [13]. In these cases, the apparently intuitive concept that “the higher the T1 values, the more severe the injury” does not always hold true, and illustrates the complexity of using T1 values alone to predict outcomes in the presence of MVO and/or IMH at 24 h post MI. Our study highlighted the differential effect of timing when using native T1 mapping in stratifying patients following STEMI. The lack of association between average T1 value of the AAR at 24 h and standard infarct characteristics at both 24 h and 6 months suggest that these imaging biomarkers may reflect distinct (but likely overlapping) tissue changes in early infarct evolution; whether T1-AAR at 24 h provides added value to standard CMR indices in infarct characterization or prediction of outcomes awaits further research.

Different imaging biomarkers have been proposed to stratify STEMI patients, including LGE myocardium and MSI [7, 16]. However, the prognostic role of these biomarkers was dependent merely on the percentage size of the LV affected and ignored the potential role of within-tissue heterogeneity. The accuracy and clinical application of acute LGE, and subsequently MSI, have been challenged [3, 17]. Acute gadolinium-based parameters overestimate the necrotic myocardium because of the increased extracellular space in the peri-infarct area, assigning potentially low risk subjects into high risk group [3, 17]. Recently, a large data-pooled meta-analysis demonstrated a significant association between mortality and MVO [18]. This relationship remained significant after adjusting for infarct size and highlighting the importance of infarct composition on long term outcomes [18]. Importantly, MVO in that analysis was quantified, on average, 3 days after STEMI when patients are typically discharged, making its application for guiding clinical decisions less useful. Moreover, the need to administer contrast agent and to wait for it to distribute within the myocardium limits the potential for application of MVO at very early time post-STEMI.

Native T1 mapping can provide a quantitative assessment of myocardial tissue composition in the setting of acute STEMI, without the need for contrast agents [4, 10]. The technique has the potential to assess severity of reperfusion injury or effectiveness of reperfusion treatment early after the event and might be used to assess measures aimed at reducing reperfusion injury.. Previous experimental work demonstrated that T1 values were related to the duration of ischaemia reflecting changes of intra- and extracellular environments within the myocardium [19]. Mechanistically, the increase in T1 values is largely related to the increase in tissue water content in response to acute ischaemic insult [10, 19]. Following STEMI, the processes that dictate infarct progression; such as myocyte death, degradation of extracellular matrix and microvascular dysfunction, are potentially detectable on T1 mapping [4, 10]. Clinical or procedural characteristics were not related to infarct severity, as measured by T1 value within the injured myocardium. Importantly, T1 mapping was performed after PCI and therefore it is not possible to delineate effects of initial infarct severity from reperfusion injury following PCI. Future studies may identify additional clinical or procedural characteristics that would influence the temporal changes in T1 values. Early characterisation may provide insights of myocardial potential of recovery, even before primary PCI in very selected population [8].

Infarct size and ejection fraction are considered as surrogates of infarct severity, nonetheless, their relationship with hyper-acute T1 value was not similar. The lack of correlation between ejection fraction and infarct size in non-large infarcts suggest a complex interaction between these two imaging biomarkers and may explain their association with T1 value [20]. IMH was also not statistically different between high and low T1 values groups. This may be related to the timing of 24 h CMR to quantify IMH as IMH has been reported to peak 3 days post STEMI [21].

### Limitations

The sample size in our study is relatively small and the implications for clinical outcomes cannot be derived from a study of 40 patients. Early CMR imaging assessment precludes high risk and hemodynamically unstable patients. Those patients were excluded from our study, nonetheless, they have already declared themselves as high risk individuals and so stratification with imaging is less pressing. CMR analysis was performed using a threshold of 2SD for AAR and 5SD for LGE. While these image post-processing approaches are endorsed by expert consensus from the Society for Cardiovascular Magnetic Resonance (SCMR) [22], the SD methodology is sensitive to a number of factors, including the chosen threshold, susceptibility to spatial variations in surface coil sensitivity and the relative signal-to-noise ratio, and whether remote myocardium is also affected by the acute myocardial injury. Additionally, infarct-size quantification may vary depending on contrast agent type, dose and timing after injection, as well as the timing early after acute MI. Quantitative mapping techniques are highly dependent on the sequence used, magnetic field strength, and CMR hardware and software parameters. Threshold-based image analysis approaches, especially for novel mapping techniques, may eliminate the need for reference ROIs, although standardization of these quantitative techniques is an active area of research. Overall, in the setting of acute MI when infarct evolution is dynamic, it is important to describe the methods of image analysis and recognise the known limitations in quantification of infarct size and AAR in this setting. Additionally, T1 mapping analysis was based on the average of voxel-derived T1 value and irrespective on presence of T1 core or MVO. This may have diminished the ability of using T1 values to assess the severity of acute myocardial injury, but it was a simple approach that does not require extensive post processing. Future efforts for advanced T1-map image analysis may facilitate more streamlined and standardized image analysis approaches suitable for large clinical outcome trials.

## Conclusion

Hyper-acute T1 values of the AAR (within 3 h post PPCI, but not 24 h) predicted a larger extent of MVO and infarct size at both 24 h and 6 months follow-up. Delaying CMR scanning for 24 h could not substitute the significant value of hyper-acute average T1 in determining infarct characteristics.

## Supplementary information

**Additional file 1.** Study population and electrocardiographic analysis.

## Data Availability

The datasets used and/or analysed during the current study are available from the corresponding author on reasonable request.

## Abbreviations

AAR: Area at risk
AUC: Area under the curve
bSSFP: Balanced steady state free precession
CAD: Coronary artery disease
CMR: Cardiovascular magnetic resonance
ECG: Electrocardiogram
EF: Ejection fraction
GRE: Gradient echo
IMH: Intramyocardial haemorrhage
IQR: Interquartile range
LGE: Late gadolinium enhancement
LV: Left ventricle/left ventricular
LVEDV: Left ventricle end diastolic volume
LVESV: Left ventricle end systolic volume
MBG: Myocardial blush grade
MI: Myocardial infarction
MSI: Myocardial salvage index
MVO: Microvascular obstruction
OxAMI: Oxford for acute myocardial infarction
PPCI: Primary percutaneous coronary intervention
PSIR: Phase sensitive inversion recovery
ROC: Receiver operator curve
ROI: Region of interest
SCMR: Society for Cardiovascular Magnetic Resonance
ShMOLLI: Shortened Modified Look-Locker Inversion recovery
STEMI: ST-segment elevation myocardial infarction
TIMI: Thrombolysis in myocardial infarction
