# Supplementary material for: Hyper-acute cardiovascular magnetic resonance T1 mapping predicts infarct characteristics in patients with ST elevation myocardial infarction
Source: J Cardiovasc Magn Reson. 2020 Jan 9;22:3. doi: 10.1186/s12968-019-0593-9 (PMC6951001; doi:10.1186/s12968-019-0593-9)
Supplement: Supplementary file 1 — Additional file 1. Study population and electrocardiographic analysis. [file 12968_2019_593_MOESM1_ESM.docx]

**Supplementary material**

*Study population*

Patients were included if they had ongoing chest pain for at least 30 minutes associated with ST-segment elevation >2 mm in at least two contiguous leads or 1 mm in two limb leads. Patients were excluded if the culprit vessel was not occluded (TIMI flow > 0), by the presence of severe hemodynamic instability, previous myocardial infarction, previous coronary artery bypass graft, pregnancy, severe renal impairment or contraindication to CMR including implanted pacemaker, defibrillator, or other metallic implanted devices or by claustrophobia. In addition, whenever CMR was not feasible within the required timeframe (for logistic reasons), patients were not recruited to the study.

*Electrocardiographic analysis*

Measurements were performed blinded to clinical and CMR measurements. The sum of pre and post-PPCI ST-segment elevation was measured at 20 msec from J-point in the leads V1–V6, I, and aVL for anterior infarction and in the leads II, III, aVF, V5, and V6 for non-anterior infarction. ST resolution (ΣSTR) was defined as complete if post PPCI ST segment sum presented a >70% reduction compared to baseline.

**Oxford Acute Myocardial Infarction (OxAMI) Study**

Keith Channon, Division of Cardiovascular Medicine, University of Oxford, UK

Robin Choudhury, Division of Cardiovascular Medicine, University of Oxford, UK

Adrian Banning, Oxford University Hospitals NHS Foundation Trust

Raj Kharbanda, Oxford University Hospitals NHS Foundation Trust

Neil Ruparelia, Division of Cardiovascular Medicine, University of Oxford, UK

Mohammad Alkhalil, Division of Cardiovascular Medicine, University of Oxford, UK

Gian Liugi De Maria, Oxford University Hospitals NHS Foundation Trust

Lisa Gaughran, Division of Cardiovascular Medicine, University of Oxford, UK

Erica Dall’Armellina, Division of Cardiovascular Medicine, University of Oxford, UK Vanessa Ferreira, Division of Cardiovascular Medicine, University of Oxford, UK

Alessansdra Borlotti, Division of Cardiovascular Medicine, University of Oxford, UK
